# Hardening Plaster Casts

**Published:** 1883-05

**Authors:** 


### HARDENING PLASTER CASTS.

When it is desirable to preserve a plaster cast, or when it is important that it should not be marred and injured by handling, it may be hardened by boiling it for half an hour in a strong solution of potash alum. If such a preparation be kept at hand, ready for use when required, it is but little trouble or labor to make models that are almost like metallic casts, and much of the annoyance and vexation of laboratory work may thereby be avoided.

Another method of hardening is to thoroughly dry the plaster and paint it over repeatedly with thin shellac varnish, drying the cast between the application of the different coats, until saturation is accomplished.

